# Primary Study for the Therapeutic Dose and Time Window of Picroside II in Treating Cerebral Ischemic Injury in Rats

**DOI:** 10.3390/ijms13032551

**Published:** 2012-02-23

**Authors:** Haitao Pei, Xi Su, Li Zhao, Hongyun Li, Yunliang Guo, Menizeng Zhang, Hui Xin

**Affiliations:** 1 Department of Emergency Neurology, Affiliated Hospital of Qingdao University Medical College, Qingdao 266003, China; E-Mails: peihtao@163.com (H.P.); hellosuxi@163.com (X.S.); honyunli@gmail.com (H.L.); 2 Institute of Integrative Medicine, Qingdao University Medical College, Qingdao 266003, China; E-Mail: 675078982@qq.com; 3 Department of Neurology, Affiliated Hospital of Qingdao University Medical College, Qingdao 266003, China; E-Mail: zmzcmd@yahoo.com.cn; 4 Department of Cardiology, Affiliated Hospital of Qingdao University Medical College, Qingdao 266003, China; E-Mail: xhqy2002@163.com

**Keywords:** picroside II, cerebral ischemia, therapeutic dose, time window, NSE, S-100, rats

## Abstract

The aim of this study was to explore the optimal therapeutic dose and time window of picroside II for treating cerebral ischemic injury in rats according to the orthogonal test. The middle cerebral artery occlusion (MCAO) models were established by intraluminally inserting a thread into middle cerebral artery (MCA) from left external carotid artery (ECA). The successful rat models were randomly divided into 16 groups according to the orthogonal layout of [*L*_16_(4^5^)] and treated by injecting picroside II intraperitoneally with different doses at various times. The neurological behavioral function was evaluated by Bederson’s test and the cerebral infarction volume was measured by tetrazolium chloride (TTC) staining. The expressions of neuron specific enolase (NSE) and neuroglial mark-protein S-100 were determined by immunohistochemisty assay. The results indicated that the optimal compositions of the therapeutic dose and time window of picroside II in treating cerebral ischemic injury were ischemia 1.5 h with 20 mg/kg body weight according to Bederson’s test, 1.0 h with 20 mg/kg body weight according to cerebral infarction volume, 1.5 h with 20 mg/kg body weight according to the expressions of NSE and S-100 respectively. Based on the principle of the minimization of medication dose and maximization of therapeutic time window, the optimal composition of the therapeutic dose and time window of picroside II in treating cerebral ischemic injury should be achieved by injecting picroside II intraperitoneally with 20 mg/kg body weight at ischemia 1.5 h.

## 1. Introduction

Cerebral ischemic reperfusion injury is a pathological process resulting from many factors, such as excitatory amino acids releasing, oxidative stress, calcium ion overloading, inflammatory reaction, apoptosis, *etc*. [1–3]. Neuron-specific enolase and neuroglial mark-protein S-100 can reflect the degree of cerebral injury [4–6]. The former study of cell culture proved that pcroside II could lessen the injury to PC12 induced by H_2_O_2_ and raise the cell survival rate [7–12]. Some animal experiments proved that picroside II could inhibit apoptosis and the expression of related inflammatory factors in ischemic penumbra after ischemic reperfusion injury [13–15]. The authors’ former experiments indicated that picroside II could reduce the degradation of the substrate, poly ADP-ribose polymerase (PARP) with catalytic activity, by inhibiting the expression toll-like receptor 4 (TLR4), nuclear transcription factor κB (NFκB), tumor necrotic factor α (TNFα) and Capase-3, and make PARP to utilize the remanent energy in the cells of ischemic penumbra to repair the nerve cells which have the reversibility of refreshing, so that to inhibit the ischemic injury leading to the apoptosis [16–23]. However, in these experiments, the rats were treated by injecting picroside II with a simple dose (20 mg/kg) at a simple time after ischemia (2 h) from tail vein. In this study, the authors aimed to explore the optimal therapeutic dose and time window of picroside II injecting intraperitoneally in cerebral ischemic injury in rats according to the design principle of orthogonal test.

## 2. Results and Discussion

### 2.1. Neurobehavioral Deficit Score

After treated by picroside II, each rat showed behavioral defect at different degree. By the analysis of software of SPSS (set α = 0.05), the probability of significant interaction of independent variable was 0.46 > 0.05 (Table 1), and there was no interaction between administering drug time and administering drug dose. From the result of analysis of variance, the significant probability of each independent variable was *P* = 0.02 < 0.05. Therefore, medication time and dose have significant influence on recovery of neurological function. All data are analyzed by the statistical mean of two-way ANOVA (analysis of variance) and LSD (least significant difference) value, the analytic results showed that there was no significant differences between administering drug at ischemia 1 h (A1) and 1.5 h (A2), and between administering drug at ischemia 2 h (A3) and 2.5 h (A4), *P* > 0.05. There was no significant deviation between administering drug doses of 5 mg/kg (B1) and 10 mg/kg (B2) and between administering drug doses of 20 mg/kg (B3) and 40 mg/kg (B4), *P* > 0.05. In addition, there was a significant differences between each other for the rest groups, *P* < 0.05, so that the better composition of the therapeutic dose and time window of picroside II were A1B3, A1B4, A2B3, A2B4. Considering the principles of medication dose minimization and therapeutic time maximization, it is presumed that A2B3 is the best composition, namely the best therapeutic time window and the best therapeutic dose of picroside II is ischemia 1.5 h with 20 mg/kg body.

### 2.2. Volume of Cerebral Infarction

After treated by picroside II, all rats showed an infarction volume at different degree in the area supplied by middle cerebral artery with TTC staining. According to the analysis with software of SPSS (set α = 0.05), the significant probability of the interaction of independent variables is 0.23 > 0.05, so there is no interaction between medication time and therapeutic dose (Tables 2 and 3). The result of ANOVA showed that the significance probability of every independent variable was *P* < 0.01, which indicated the medication time and therapeutics dose of picroside II influenced on cerebral infarction volume significantly. All data are analyzed by the statistical mean of two-way ANOVA and LSD value. It is concluded that there was significant deviation between each other of medication time (*P* < 0.05), no significant deviation between therapeutic dose 20 mg/kg (B3) and 40 mg/kg (B4) (*P* > 0.05), and there was a significant deviation between the rest combination, *P* < 0.05. Therefore, the better combination of medication time and therapeutic dose were A1B3 or A1B4. According to the principles of the minimization of medication dose and maximization of therapeutic time, it is presumed that A1B3 is the best composition group, *i.e.*, the best therapeutic time window and the best therapeutic dose of picroside II is ischemia 1 h with 20 mg/kg body.

### 2.3. Expression of NSE Protein

The immunohistochemisty staining shows that NSE in brain tissue expressed at different degrees, and mainly in cytoplasm with yellow or brown colors. The significance probability of the interaction of independent variables (the number of positive cells) is 0.78 > 0.05, so there is no interaction between medication time and therapeutics dose (Tables 4 and 5). The result of ANOVA showed that the significance probability of every independent variable is less than 0.01, which proved that medication time and therapeutic dose of picroside II could significantly influence the expression of NSE. All data are analyzed by the statistical mean of two-way ANOVA and LSD value, it is concluded that there was a significant deviation of NSE expression between each medication time (*P* < 0.05), while no significant deviation between 20 mg/kg (B3) and 40 mg/kg (B4) (*P* > 0.05), but there was significant deviation between the rest therapeutic dose combination (*P* < 0.05). So the better combination of medication time and therapeutics dose is A1B3 or A1B4. Considering the minimization of medication dose and maximization of therapeutic time, it is presumed that A1B3 is the best combination, namely the best therapeutic time window and the best therapeutic dose of picroside II is ischemia 1.0 h with 20 mg/kg.

### 2.4. Expression of S-100 Protein

The immunohistochemial stain shows the expression of S-100 in brain tissue was at different degree and mainly in cytoplasm. The significance probability of the interaction of independent variables (the number of positive cells) is 0.35 > 0.05, so there is no interaction between medication time and therapeutic dose (Tables 6 and 7). The result of ANOVA showed that the significance probability of every independent variable is smaller than 0.01, which suggested that the medication time and therapeutic dose of picroside II could significantly influence on S-100 expression. All data are analyzed by the statistical mean of two-way ANOVA and LSD value. It is concluded that there was no significant deviation of S-100 expression between ischemia 1 h (A1) and ischemia 1.5 h (A2), also ischemia 1.5 h (A2) and 2 h (A3) (*P* > 0.05), while there was a significant deviation between the rest combination (*P* < 0.05). Although no significant deviation between therapeutic dose 5 mg/kg (B1) and 10 mg/kg (B2), or 20 mg/kg (B3) and 40 mg/kg (B4) (*P* > 0.05), there was a significant deviation between the rest combination (*P* < 0.05). So the better combination of medication time and therapeutic dose is A1B3 or A1B4 or A2B3 or A2B4. Considering the minimization of medication dose and maximization of therapeutic time, it is presumed that A2B3 is the best combination, namely the best therapeutic time window and the best therapeutic dose of picroside II is ischemia 1.5 h with 20 mg/kg body.

### 2.5. Discussion

Orthogonal layout can balance sampling in the changing range of variable factors, and can enhance the representation of each test with minimum animal number and test times. Orthogonal layout has characteristics of balanced scattering, which satisfies some prerequisites of a comprehensive test, shortened test cycle and elevated test efficiency to achieve a better test aim. In this paper, the authors applied orthogonal layout to design roundly, compared synthetically with statistical analysis to obtain a better therapeutic schedule to get the best treatment effectiveness with a small number of tests. For statistical analysis of the orthogonal test, we chose 2-way ANOVA.

In this experiment, the neurological behavioral function was evaluated by Bendeson’s test to judge the therapeutic effect of picroside II on cerebral ischemic injury, TTC staining was observed and calculate the cerebral infarction volume to indicate the severity of cerebral ischemic injury. The neuroglial mark-protein S-100 [24–26] and the neuron-specific enolase (NSE) are sensitive indexes to evaluate cerebral ischemic injury [27,28]. NSE is a kind of acidic soluble protein only existing in nervous tissue. According to their three subunits with different immunity, enolases are divided into five kinds of isozymes as follows: type αα, ββ, γγ, αβ and αγ, of which type γγ exists specially in nerve cells and being named as NSE. Enolases participate in the metabolism of glucolysis, catalysis α-phosphoglyceric acid into enolphosphopyruvate. S-100 protein is an acidity calcium-binding protein, not simple protein but boodle of micromolecule protein with α and β subunits, of which the S-100 with β-subunit particularly exist in nerve tissue, and mainly in gliacyte. S-100 is a kind of intercellular calcium-receptor protein and regulates energy metabolism, promotes axon growth and gliocyte proliferation, stabilizes internal environment of calcium ion, *etc*. In the physiological circumstances, the contents of NSE and S-100 in brain tissue and serum are seldom and increase after brain ischemic injury to release in to the blood circulation because of cerebral ischemia injuring the blood-brain barrier. NSE and S-100 are neurochemistry markers reflecting the severity of brain tissue damage [25], so it could reflect the neuroprotective effects of picroside II on cerebral ischemia by detecting the contents of NSE and S-100 in brain tissue and serum.

In this experiment, the authors designed four time points at 1 h, 1.5 h, 2 h and 2.5 h after brain ischemic injury, and inject intraperitoneally picroside II with four therapeutic doses of 5 mg/kg, 10 mg/kg, 20 mg/kg and 40 mg/kg. The experiment was carried out according to orthogonal table of [*L*_16_(4^5^)] to explore the best therapeutic dose and best time window of picroside II in treating cerebral ischemic injury by neurological faction scale, TTC staining to measure cerebral infarction volume and the expressions of NSE and S-100, *etc*. The results indicated that there is a significant difference between administering drug time and therapeutic drug dose of picroside II in influencing the multiple comparison of statistical analysis showed that the best combination is not with accordant by different indexes. Considering minimization of medication dose and maximization of therapeutic time window, it is suggested the best choose of A1B3 composition, or the best therapeutic time window is at 1.0 h after ischemia and the best therapeutic dose of picroside II is 20 mg/kg. Because the mechanism of cerebral ischemic injury is very complicated and only four indexes was observed in this experiment, the results could not possibly all be right. So the effect and mechanism of picroside II and the golden evaluating indexes need to be further studied in further experiments.

## 3. Experimental Section

### 3.1. Animal Model of MCAO

Adult healthy male *Wistar* rat, SPF grade, weight 240–260 g, were supplied by the Experiment Animal Center of Qingdao Drug Inspection Institute (SCXK (LU) 20090100). The local legislation for ethics of experiment on animals and guidelines for the care and use of laboratory animals were followed in all animal procedures. This experiment was approved by the Ethics Committee of Qingdao University Medical College (No. QUMC 2011-09). All animals were acclimatized for 7 days and allowed free access to food and water in a room temperature (23 ± 2 °C) and humidity-controlled housing with natural illumination and absolute diet at preoperative 12 h before operation. The rats were anesthetized by injecting intraperitoneally 100 g/L chloral hydrate (300 mg/kg) and fixed in supine position to conduct aseptic operation. The middle cerebral artery occlusion (MCAO) model was established by inserting intraluminaly a monofilament suture from the left external-internal carotid artery (ECA-ICA) into the middle cerebral artery (MCA) for cerebral ischemia [29]. Core body temperature was checked by a rectal probe and maintained at 36–37 °C using a homeothermic blanket control unit (Qingdao Apparatus, China) during and after the surgery operation. The successful model rats showed left Horner’s sign, right forelimb flexing and circling rightward as running.

### 3.2. Orthogonal Experimental Design

Sixteen successful MCAO rat models were internalized into the experiment and divided randomly according to the principle of orthogonal experimental design of [*L*_16_(4^5^)] consisting of two impact factors with four impact levels (Table 8). The impact factor A is the therapeutic time widow designed four levels: 1.0 h, 1.5 h, 2.0 h, 2.5 h after ischemia. The impact factor B is the therapeutic drug dose which has four levels as follows: 5 mg/kg, 10 mg/kg, 20 mg/kg and 40 mg/kg body weight (Table 5).

### 3.3. Treatment Methods

Picroside II (Tianjin Kuiqing Medical Technology Co., Ltd., CAS No: 39012-20-9, purity >98%) was diluted into 1% solution with 0.1 mol/L PBS and injected intraperitoneally according to the corresponding designed doses in the orthogonal layout [*L*_16_(4^5^)]. After treatment for 24 h, the rats were killed to detect the corresponding indexes.

### 3.4. Observation Indexes

#### 3.4.1. Neurological Defect Test

After treatment with picroside II, the neurological behavioral function scales were performed by Bederson’s test [30] in each rats. The higher the score, the more severe the neurological function defect. Score 0: no behavioral deficiency; Score 1: forelimb buckling (the tail lifting-suspension test positive); Score 2: lateral thrust resistance decreased (lateral thrust test positive) and forelimb buckling, no circling; Score 3: the same as midrange behavior and circling spontaneously.

#### 3.4.2. The Cerebral Infarction Volume

**Figure 1:**
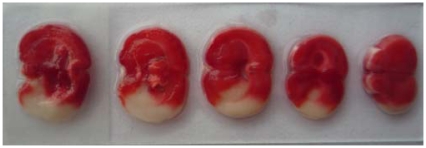
The cerebral infarction volume. The normal brain tissues showed red and infarct tissue white by TTC staining.

After treatment with picroside II, all rats were anesthetized with 100 g/L chloral hydrate (300 mg/kg) and then the brain was take out completely to be cut five coronal sections of 2 mm thickness backward from the Bregma. Five coronal sections were put into 20 g/L TTC phosphate buffer at 37 °C and then fixed in 40 g/L poly-formaldehyde. The normal brain tissues showed red and infarct tissue is white (Figure 1). The infarct volume is determined by Adobe PhotoShop CS after taking a photograph, and expressed as the percentage of the brain infarct size and homonymy brain hemisphere (%).

#### 3.4.3. Immunohistochemisty

**Figure 2:**
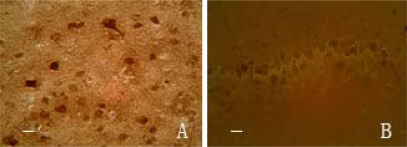
The expressions of NSE in cortex (**A**) and S-100 in hippocampus (**B**). The immunochemical positive cells showed brown granules in cytoplasm, bar 25 μm.

After treatment with picroside II, total of rats were anesthetized with 100 g/L chloral hydrate (300 mg/kg) and fixed with 40 g/L poly-formaldehyde from cardiac perfusion to take out the brain completely. The brain was dehydrated by gradient ethanol, cleared by xylene, embedded in paraffin and sliced backward from optic chiasma into pieces of thickness 5 μm. Then adhered those slices to the sections prepared with poly-l-lysine, and finally stored at 4 °C. Rabbit anti-rat NSE and S-100 moloclonal antibodies, SABC immunohistochemisty kit and DAB chromogenic liquor were provided by Boster Biological Company, Wuhan, China. The paraffin section were deparaffinized according to directions, then colored by DAB and observed under light microscope to count the positive cells with brown granules in cytoplasm. And the slides with the addition of 0.01 mmol/L PBS (containing 1:200 non-immunity animal serum), instead of primary antibody, showed no response. Five serial section (1mm backward from optic chiasma) of each rat were randomly chosen and observed five visual fields of cortical area under light microscope (400 times) to count the number of positive cells of every visual fields and expressed as mean ± standard error (*χ̄* ± s) (Figure 2).

### 3.5. Statistical Analysis

SPSS 17.0 software was used for data statistical analysis (Table 9). According to the result, multi-group comparison was made by analysis of orthogonal test whether different level of administrating time and therapeutic dose had significant deviation or not, and whether their interaction on each detected index had significant deviation or not, meanwhile to explore the best therapeutic drug dose and the therapeutic time window. Values were considered to be significant when *P* was less than 0.05.

## 4. Conclusions

This study suggested the optimal composition of the therapeutic dose and time window of picroside II in treating cerebral ischemic injury should be injecting picroside II intraperitoneally with 20 mg/kg body weight at ischemia 1.5 h after considering the minimization of medication dose and maximization of therapeutic time.

## Figures and Tables

**Table 1:** Analysis of variance about neurological function defect.

Variation source	SS	df	MS	*F*	*P*
Time	3.187	3	1.06	17	0.02
Dose	3.187	3	1.06	17	0.02
Time*Dose	0.188	3	0.63	1	0.45

Error	0.375	6	0.63		

The significant probability of independent variable time was *P* = 0.02 < 0.05; the significant probability of independent variable dose was *P* = 0.02 < 0.05; the significant probability of independent variable interaction was *P* = 0.45 > 0.05.

**Table 2:** The infarction volume of tetrazolium chloride (TTC) staining.

Drug Dose	Ischemia 1.0 h	Ischemia 1.5 h	Ischemia 2.0 h	Ischemia 2.5 h
5 mg/kg	69.05	73.00	73.40	75.50
10 mg/kg	66.20	71.50	72.10	74.30
20 mg/kg	64.90	69.30	71.60	73.90
40 mg/kg	64.50	68.90	70.80	72.00

**Table 3:** ANOVA of cerebral infraction volume.

Variation source	SS	df	MS	*F*	*P*
Time	130.46	3	43.49	150.30	0.00
Dose	30.32	3	10.11	34.93	0.00
Time*Dose	1.67	3	0.56	1.92	0.23

Error	1.74	6	0.29		

The significant probability of independent variable time was *P* = 0.00 < 0.05; the significant probability of independent variable dose was *P* = 0.00 < 0.05; the significant probability of independent variable interaction was *P* = 0.23 > 0.05.

**Table 4:** The expression positive cells of neuron specific enolase (NSE).

Drug Dose	Ischemia 1.0 h	Ischemia 1.5 h	Ischemia 2.0 h	Ischemia 2.5 h
5 mg/kg	36.20	38.33	39.90	41.00
10 mg/kg 35.00	36.90	37.55	38.00	
20 mg/kg	34.33	35.55	36.80	37.60
40 mg/kg	34.20	35.60	36.20	37.20

**Table 5:** ANOVA of positive cells of NSE.

Variation source	SS	df	MS	*F P*	
Time	27.497	3	9.17	39.39	0.00
Dose	22.928	3	7.64	32.85	0.00
Time*Dose	0.253	3	0.08	0.36	0.78

Error	1.649	6	0.23		

The significant probability of independent variable time was *P* = 0.00 < 0.05; the significant probability of independent variable dose was *P* = 0.00 < 0.05; the significant probability of independent variable interaction was *P* = 0.78 > 0.05.

**Table 6:** The expression positive cells of S-100.

Drug Dose	Ischemia 1.0 h	Ischemia 1.5 h	Ischemia 2.0 h	Ischemia 2.5 h
5 mg/kg	43.88	45.00	45.90	46.00
10 mg/kg	40.90	44.50	45.10	45.70
20 mg/kg	37.06	38.25	40.00	44.60
40 mg/kg	36.03	37.60	39.00	44.00

**Table 7:** ANOVA of positive cells of S-100.

Variation source	SS	df	MS	*F*	*P*
Time	66.09	3	22.03	11.36	0.01
Dose	106.18	3	35.39	18.26	0.01
Time*Dose	7.69	3	2.56	1.32	0.35

Error	19.00	6	1.94		

The significant probability of independent variable time was *P* = 0.01 < 0.05; the significant probability of independent variable dose was *P* = 0.01 < 0.05; the significant probability of independent variable interaction was *P* = 0.35 >0.05.

**Table 8:** Orthogonal experimental design of [*L*_16_(4^5^)].

Therapeutic dose	Ischemia 1.0 h (A1)	Ischemia 1.5 h (A2)	Ischemia 2.0 h (A3)	Ischemia 2.5 h (A4)	Therapeutic dose
5 mg/kg (B1)	1.0 × 5	1.5 × 5	2.0 × 5	2.5 × 5	5 mg/kg (B1)
10 mg/kg (B2)	1.0 × 10	1.5 × 10	2.0 × 10	2.5 × 10	10 mg/kg (B2)
20 mg/kg (B3)	1.0 × 20	1.5 × 20	2.0 × 20	2.5 × 20	20 mg/kg (B3)
40 mg/kg (B4)	1.0 × 40	1.5 × 40	2.0 × 40	2.5 × 40	40 mg/kg (B4)

**Table 9:** [*L*_16_(4^5^)] orthogonal layout and the results of infarction volume.

Test NO.	Rank NO.					Infarction volume

	A	B	C	D	E	
1	1	1	1	1	1	69.05
2	1	2	2	2	2	66.20
3	1	3	3	3	3	64.90
4	1	4	4	4	4	64.50
5	2	1	2	3	4	73.00
6	2	2	1	4	3	71.50
7	2	3	4	1	2	69.30
8	2	4	3	2	1	68.90
9	3	1	3	4	2	73.40
10	3	2	4	3	1	72.10
11	3	3	1	2	4	71.60
12	3	4	2	1	3	70.80
13	4	1	4	2	3	75.50
14	4	2	3	1	4	74.30
15	4	3	2	4	1	73.90
16	4	4	1	3	2	72.00

I	264.65	290.95	284.15			1132.95
II	282.70	284.10	283.90			
III	287.90	279.70	281.50			
IV	295.70	276.20	281.40			
SS	130.46	30.32	1.677			
